# Grape seed proanthocyanidins ameliorate pancreatic beta-cell dysfunction and death in low-dose streptozotocin- and high-carbohydrate/high-fat diet-induced diabetic rats partially by regulating endoplasmic reticulum stress

**DOI:** 10.1186/1743-7075-10-51

**Published:** 2013-07-21

**Authors:** Ye Ding, Zhaofeng Zhang, Xiaoqian Dai, Yanfei Jiang, Lei Bao, Yujie Li, Yong Li

**Affiliations:** 1 Department of Nutrition and Food Hygiene, School of Public Health, Peking University, Beijing, PR, China

**Keywords:** Grape seed proanthocyanidins, Pancreatic beta-cell failure, Endoplasmic reticulum stress, Insulin, High-carbohydrate/high-fat diet, Streptozotocin, Type 2 diabetes mellitus

## Abstract

**Background:**

It is increasingly being realized that failure of pancreatic beta cells to secrete enough insulin to adequately compensate for obesity and insulin resistance is the primary defects of type 2 diabetes mellitus (T2DM). Pancreatic beta cells possess a highly developed and active endoplasmic reticulum (ER), reflecting their role in folding, export and processing of newly synthesized insulin. ER stress-induced pancreatic beta-cell failure is a novel event in the pathogenesis of T2DM. Some studies with antioxidants indicated a beneficial impact on ER stress. Our previous study found that strong antioxidants, grape seed proanthocyanidins (GSPs), ameliorated ER stress to protect skeletal muscle from cell death in type 2 diabetic rats. The present study continued to investigate the effect of GSPs on beta-cell failure and ER stress in diabetic pancreas.

**Methods:**

Male Sprague–Dawley rats made type 2 diabetic with 2 injections of 25 mg/kg streptozotocin and 8 weeks of the high-carbohydrate/high-fat diet were fed a basal diet with or without GSPs administration for 16 weeks. Oral glucose tolerance, plasma glucose, serum insulin and the score of beta-cell function were measured. Morphological observation was performed by light and electron microscopic analyses. Islet cell apoptosis was determined by terminal deoxynucleotidyl transferase-mediated dUTP biotin nick end labeling staining. Additionally, the level of insulin and the expression of ER stress markers in pancreatic islets were also studied using immunohistochemical staining.

**Results:**

After 16 weeks treatment, the score of beta-cell function and the abnormal oral glucose tolerance of diabetic rats were partially reversed by GSPs treatment. The efficacious effect of GSPs was also manifested in the amelioration of pancreatic damage and ER dilatation by microscopic analyses. Moreover, GSPs treatment increased normal insulin content and decreased the number of apoptotic cells in diabetic islets. Importantly, GSPs treatment partially alleviated ER stress by decreasing some ER stress markers.

**Conclusion:**

These findings suggest that GSPs might have auxiliary therapeutic potential for pancreatic beta-cell dysfunction and death in T2DM.

## Background

Modern lifestyles, with increased caloric consumption and reduced physical activity, have dramatically increased the rates of obesity-associated disease conditions, including type 2 diabetes mellitus (T2DM). It was estimated that approximately 366 million people (aged 20–79) worldwide had diabetes in the year 2011; with T2DM accounting for 90-95% of all diagnosed cases [1]. Numerous studies show that insulin resistance, often associated with obesity and physical inactivity, precedes the development of hyperglycemia in subjects that eventually develop T2DM [2,3]. However, it is increasingly being realized that failure of pancreatic beta cells to secrete enough insulin to adequately compensate for obesity and insulin resistance is the primary defects of T2DM [4,5]. Therefore, effective therapeutic strategies to prevent or delay the development of pancreatic beta-cell dysfunction and death may be desirable for the control of this disorder.

Pancreatic beta cells possess a highly developed and active endoplasmic reticulum (ER), reflecting their role in folding, export and processing of newly synthesized insulin [6]. Certain conditions, such as high lipid load, hyperglycemia, oxidative stress, excessive Ca^2+^ release from ER stores, or misfolded mutant insulin proteins, will disrupt ER homeostasis, resulting in an adaptive unfolded protein response (UPR), which aims to restore ER folding capacity and mitigate stress [7]. One of the most described mechanisms of UPR activation is the competition model, in which the ER chaperone protein glucose regulated protein 78 (GRP78) plays an essential role in the activation of different ER stress transducers [7]. However, under conditions of severe and prolonged ER stress, the UPR is unable to restore normal cellular function. Subsequently, cell death is triggered. This effect is mediated in part by increased expression of the transcription factor C/EBP homologous protein (CHOP) and activities of Jun N terminal kinase (JNK) and Caspase-12 [8]. Accumulating evidence based on *in vivo* and *in vitro* studies also has demonstrated that ER stress is a novel causative factor of pancreatic beta-cell dysfunction and death in the pathogenesis of T2DM [9-11].

Some studies showed that antioxidants (e.g. the heavy metal scavenger antioxidant metallothionein, and antioxidant N-acetylcysteine) had a beneficial impact on ER stress [12,13], indicating that the use of antioxidants offer the possibility for improvement of ER stress and beta-cell dysfunction in T2DM. In recent years, natural dietary components are being pursued as alternatives to pharmaceutical interventions. Grape seed proanthocyanidins (GSPs), which are derived from grape seeds, refer to a group of proanthocyanidins mostly containing dimers, trimers and other oligomers of catechin and epicatechin and their gallic acid esters. Interestingly, the *in vitro* antioxidative activities of GSPs were found to be much stronger than that of vitamin C and vitamin E, singly and in combination [14,15]. Moreover, previous animal studies based on type 1 diabetes mellitus reported that GSPs exerted anti-hyperglycemic property [16,17]. Among these few studies, GSPs was reported to ameliorate pancreatic damage by alleviation of oxidative stress [16]. Our previous study found that GSPs ameliorated ER stress to protect skeletal muscle from cell death in a type 2 diabetic model [18]. However, the protective effect of GSPs on pancreatic damage of T2DM and the relevant mechanisms of ER stress also need further elucidation.

In the present study, we used low dose streptozotocin (STZ) and a high-carbohydrate/high-fat diet induced type 2 diabetic rats to investigate whether long-term GSPs administration would result in the improvement of pancreatic beta-cell dysfunction and death and to check whether this protective effect of GSPs would be, in part, due to downregulation of ER stress.

## Materials and methods

### Animals

The Institutional Animal Care and Use Committee of Peking University approved the protocols before starting. 80 male Sprague–Dawley rats (180–200 g) were purchased from Animal Service of Health Science Center (Peking University) and housed 2 per cage in this center with 12 h light-12 h dark cycles (light time began at 7:30 AM) under controlled humidity (60 ± 5%) and temperature (25 ± 1°C). All animal care and experimental procedures were in accordance with the Guide for the Care and Use of Laboratory Animals (NIH publication No. 85–23, 1985).

### Reagents

Basal diet (AIN-93G diet) and the high-carbohydrate/high-fat diet (66% basal diet, 15% lard, 10% plantation white sugar, 6% casein and 3% yolk powder) were produced by Beijing Keao Xieli Co. Ltd. (Beijing, China). GSPs (Lot No: 1003007–24) were purchased from Jianfeng Natural Products Co. Ltd. (Tianjing, China). The proanthocyanidin content was 96.64% while analyzed using HPLC with gas chromatography–mass spectrometry detection. They contained 6.1% catechin, 6.78% epicatechin, 55.59% dimeric forms, 11.91% trimeric forms, 6.55% tetrameric forms and small amounts of other polymeric forms. STZ and proteinase K were from Sigma-Aldrich (St. Louis, Missouri, USA). In situ cell death detection, [terminal deoxynucleotidyl transferase-mediated dUTP biotin nick end labeling (TUNEL) assay] kit was purchased from Roche Molecular Biochemicals (Mannheim, Germany). The insulin antibody was from Cell Signaling Technology (Danvers, Massachusetts, USA). The antibodies for GRP 78, CHOP, phosphorylated JNK and Caspase-12 were obtained from Santa Cruz Biotechnology (California, USA). 4’,6-diamidino-2-phenylindole (DAPI), fluorescein isothiocyanate (FITC)-labeled secondary antibody and the immunohistochemistry kit were from Beijing Zhongshan Golden Bridge Biotechnology Co. Ltd. (Beijing, China). All other common chemicals were of analytical reagent grade.

### Experimental protocol

**Figure 1:**
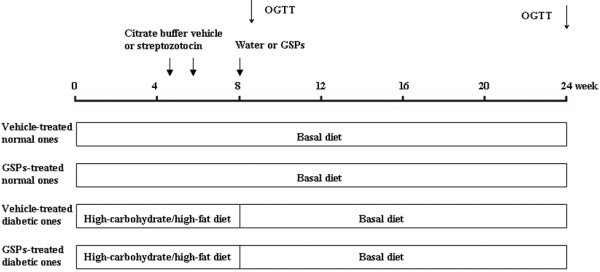
**Schematic representation of the experimental procedures.** The present study was performed in Sprague–Dawley rats made diabetic with low dose streptozotocin and a high-carbohydrate/high-fat diet. GSPs: grape seed proanthocyanidins; OGTT: oral glucose tolerance test.

Rats were acclimatized to new environment for 1 week, and were then randomly divided into 3 groups. Group 1 (n = 12, normal control) and group 2 [n = 12, 250 mg/kg body weight (BW) GSPs control] were both fed the basal diet. Rats in group 3 (n = 56) were induced by 2 injections of 25 mg/kg BW STZ and 8 weeks of the high-carbohydrate/high-fat diet as described previously [18]. Rats with plasma glucose levels between 250 mg/dl and 400 mg/dl at 2 weeks post STZ injection were considered suitable and only uniformly diabetic rats were used in the next experiments. The diabetic rats were randomly divided into 4 groups (n = 12 each), including diabetic control group and 3 GSPs intervention groups (125, 250, and 500 mg/kg BW respectively). Then, the normal and diabetic control groups were given water, while the other four groups were administered GSPs by stomach tube. During the following 16 weeks, all groups were allowed free access to the basal diet (Figure 1).

### Oral glucose tolerance test (OGTT)

The OGTT procedure was performed at the end of 1 and 16 week after induction of diabetes. Rats were food restricted and were given only water to drink for 6 h. Blood samples for plasma glucose were then collected from snipped tails by tail milking at 0, 30, 60, and 120 min after administration of D-glucose (20% solution; 2 g/kg BW) by stomach tube.

### Measurement of serum parameters

Sixteen weeks after induction of diabetes, plasma glucose and serum insulin were determined as described previously [18]. Homoeostasis model assessment (HOMA) of beta-cell function (HOMA-B) was calculated by the HOMA method using the following equations [19]: HOMA-B = (20 × fasting insulin (μIU/ml)) / (fasting glucose (mmol/l) – 3.5).

### Treatment of pancreas tissue

Following blood collection, rats were sacrificed by cervical dislocation. Pancreas were carefully excised, cleared of fat, and rinsed in ice-cold saline. After removing the excess water on the surface with filter paper, pancreas was weighed and pancreas/BW ratio was evaluated. Then part of pancreas was fixed in 4% paraformaldehyde for hematoxylin/eosin (HE), TUNEL and immunohistochemical staining. In addition, a small portion of the same pancreas region from each group was immersed overnight in 2.5% glutaraldehyde (pH 7.4) in 0.1 mol/l phosphate buffered saline (PBS) at 4°C.

### Light and electron microscopy

For light microscopy, the fixed tissue samples were dehydrated through a graded ethanol series, embedded in paraffin and cut into 7 μm-thick sections with HE stain using a routine protocol. The stained sections were then observed from × 100 to × 400 magnifications. For electron microscopy, pancreas were removed from 2.5% glutaraldehyde and adequately washed in 0.1 mol/l PBS. Blocks (approximately 1 mm wide, 2 mm long and 1 mm thick) were post-fixed in 1% osmium tetroxide, dehydrated through a graded series of ethanol, and embedded in Epon 812. After being stained with toluidine blue, suitable areas of sections for ultrastructural study were chosen. Then microsections were cut and mounted on a copper grid. All the sections were stained with 4% uranyl acetate and Reynold’s lead citrate. The ultrastructure of pancreatic beta cells was checked from × 4000 to × 12000 magnification using transmission electron microscope. Two independent pathologists performed the morphological observation in a blinded fashion.

### Detection of apoptosis

Paraffin sections were deparaffinized in xylene, downgraded in alcohol grades (100, 95, 85 and 70%), washed with PBS and treated with proteinase K (2 μg/ml) for digestion. Islet cell apoptosis was determined by TUNEL staining according to the manufacturer’s protocols. The insulin antibody and FITC-labeled secondary antibody were used to probe insulin. DAPI was used to visualize nuclei. The slides were then visualized by fluorescence microscope. Apoptotic cells exhibited strong nuclear red fluorescence. Frequency of pancreatic islet cell apoptosis was expressed as events per islet.

### Immunohistochemistry

Paraffin sections were deparaffinized, hydrated, and steamed in citrate buffer for 5 min for antigen retrieval. Endogenous peroxidase activity was inhibited using 3% hydrogen peroxide in methanol for 10 min. The sections were then incubated following the instructions in the commercial manual. Briefly, sections were blocked with protein-blocking agent followed by incubation with primary antibodies (1:200) at 4°C overnight. Then they were incubated in turn with biotinylated secondary antibodies, with streptavidin peroxidase reagent, and with 3, 3-diaminobenzidine for color development. Counterstaining was carried out with hematoxylin. Each step was separated by careful washings in PBS buffer. Slides were then analyzed from × 100 to × 400 magnifications by two blinded pathologists under a light microscope. Image-Pro Plus 6.0 software was used to assess quantitative values.

### Statistical analysis

Statistical analysis was performed using SPSS (version 13.0). All data were compared by one-way ANOVA analysis, followed by LSD (equal variances assumed) or Dunnett’s T3 (equal variances not assumed) for post-hoc test between multiple groups. Values of P < 0.05 were considered significant.

## Results

### Effect of GSPs on BW and food consumption

The number remaining alive at the end of the study in the 6 groups was 12, 12, 12, 11, 12 and 12 respectively of normal control, GSPs control, diabetic control and 3 GSPs intervention groups. As shown in Table 1, there were no differences in BW and food consumption between normal and GSPs control groups. As expected, the level of BW was lower in diabetic control rats than that in normal control ones at the end of the study (P < 0.001). Additionally, food consumption was greater in diabetic rats than that in normal control rats throughout the study period (P < 0.05 for each). However, the GSPs treatment slightly increased BW and decreased food consumption in a dose-dependent manner.

**Table 1:** Body weight (BW), food intake, homoeostasis model assessment of beta-cell function (HOMA-B), pancreas/BW ratio and the frequency of apoptotic islet cells in normal and diabetic rats after 16 weeks of GSPs treatment

**Parameter**	**Normal rats**		**Diabetic rats**			
	**Vehicle**	**GSPs (mg/kg BW)**	**Vehicle**	**GSPs (mg/kg BW)**		
		**250**		**125**	**250**	**500**
Initial BW (g)	459.85 ± 6.93	466.73 ± 7.17	441. 06 ± 11.05	453.72 ± 13.99	437.92 ± 9.71	454.30 ± 10.34
Final BW (g)	584.29 ± 13.44 ^#^	544.63 ± 14.88 ^#^	417.88 ± 9.75 ^*^	436.38 ± 9.00 ^*^	447.57 ± 12.74 ^*^	446.63 ± 10.57 ^*^
Initial food intake (g/day)	19.43 ± 0.83 ^#^	17.77 ± 1.32 ^#^	30.30 ± 1.03 ^*^	29.93 ± 1.09 ^*^	29.15 ± 1.69 ^*^	29.81 ± 0.44 ^*^
Final food intake (g/day)	23.88 ± 1.87 ^#^	20.31 ± 0.73 ^#^	38.83 ± 1.64 ^*^	37.31 ± 1.65 ^*^	34.30 ± 1.46 ^*^	30.74 ± 0.23 ^*^
HOMA-B	176.34 ± 7.88 ^#^	228.63 ± 10.63 ^* #^	35.61 ± 1.62 ^*^	47.77 ± 3.06 ^*^	46.76 ± 1.54 ^*^	51.51 ± 2.91 ^* #^
Pancreas weight (g)	1.33 ± 0.03 ^#^	1.31 ± 0.04 ^#^	1.16 ± 0.04 ^*^	1.19 ± 0.07 ^*^	1.20 ± 0.04	1.22 ± 0.04
Pancreas/BW ratio (‰)	2.27 ± 0.09 ^#^	2.40 ± 0.10 ^#^	2.84 ± 0.15 ^*^	2.73 ± 0.10 ^*^	2.69 ± 0.11 ^*^	2.74 ± 0.09 ^*^
Apoptotic cells/islet	1.75 ± 0.37 ^#^	1.58 ± 0.31 ^#^	8.92 ± 0.87 ^*^	7.75 ± 0.77 ^*^	7.42 ± 0.79 ^*^	5.36 ± 0.54 ^* #^

BW and food intake were measured once a week after induction of diabetes. The frequency of apoptotic islet cells was evaluated by averaging the number of TUNEL-positive cells in approximately 50 islets from each group. The other data were means ± SEM of 10 rats of each group.

* Designated statistically significant difference from vehicle-treated normal rats, P < 0.05, # designated statistically significant difference from vehicle-treated diabetic rats, P < 0.05.

### Effect of GSPs on glucose and insulin metabolism parameters

As described previously [16], diabetic rats showed significant increases in plasma glucose and serum insulin when compared with normal control rats (P < 0.05 for each). The 500 mg/kg BW GSPs, however, showed a significant improvement in plasma glucose level (P = 0.024) (data not shown). Additionally, HOMA-B, which is used to quantify beta-cell function, was higher in GSPs control groups than that in normal control rats (P < 0.05). As expected, STZ- and high-carbohydrate/high-fat diet treatment significantly decreased the score of HOMA-B (P < 0.001), whereas the 500 mg/kg BW GSPs treatment slightly increased this parameter (P < 0.05) (Table 1).

### Effect of GSPs on oral glucose tolerance

**Figure 2:**
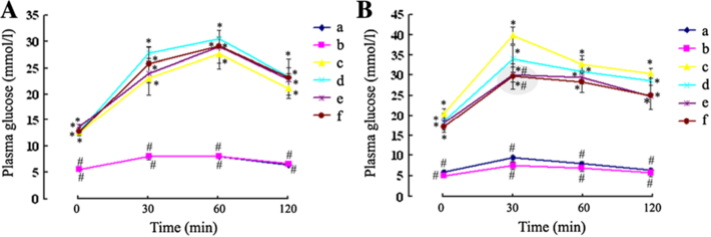
**Effect of GSPs on oral glucose tolerance in normal and diabetic rats.** Plasma glucose response during the oral glucose tolerance test procedure in normal and diabetic rats was determined at the end of 1 (**A**) and 16 (**B**) week after induction of diabetes. Values were obtained for each group of 6 animals. Group a = vehicle-treated normal rats; group b = GSPs (250 mg/kg BW)-treated normal rats; group c = vehicle-treated diabetic rats; group d = GSPs (125 mg/kg BW)-treated diabetic rats; group e = GSPs (250 mg/kg BW)-treated diabetic rats; and group f = GSPs (500 mg/kg BW)-treated diabetic rats. * P < 0.05 versus data from vehicle-treated normal rats, and # P < 0.05 versus data from vehicle-treated diabetic rats at the indicated times, respectively.

The two OGTTs were performed at the end of 1 and 16 week after induction of diabetes. The initial OGTT (Figure 2A) showed significant increases in basal, 30-, 60-, and 120-min plasma glucose values in diabetic groups when compared with normal control group (P < 0.05 for each). Data from the second OGTT were similar (Figure 2B), with significantly increased plasma glucose values observed at all time points in diabetic groups (P < 0.05 for each). Interestingly, 30-min plasma glucose values were significantly lowered in both 250 and 500 mg/kg BW GSPs-treated diabetic groups during the second OGTT when compared with diabetic control group (P = 0.022 and P = 0.016, respectively).

### Effect of GSPs on pancreas weight and pancreas/BW ratio

As shown in Table 1, there were no significant differences in pancreas weight and pancreas/BW ratio between normal and GSPs control groups. Pancreas weight of diabetic rats was significantly decreased when comparing with that of normal control ones (P = 0.009). However, pancreas/BW ratio of diabetic rats was greater than that of normal control rats (P < 0.05). This increase was almost totally explained by the decrease in BW. Regretfully, administration of GSPs had no obvious effects on pancreas weight and pancreas/BW ratio of diabetic rats.

### Effect of GSPs on pancreatic histopathology

**Figure 3:**
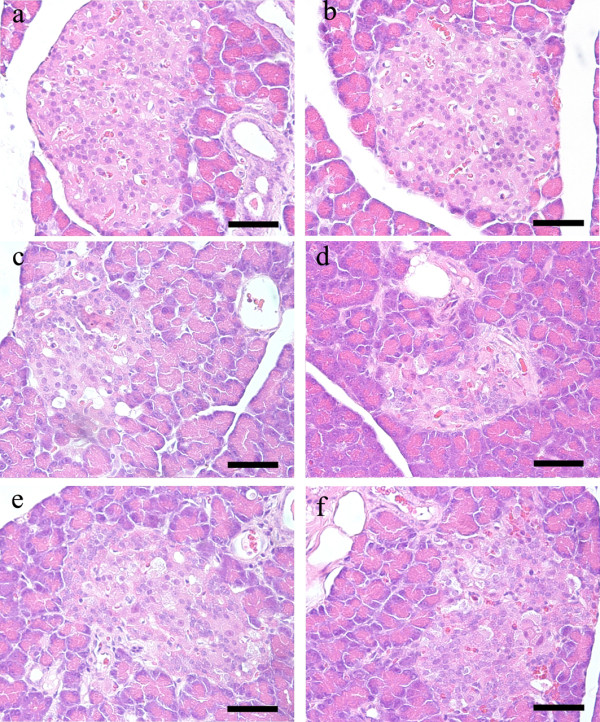
**Effect of GSPs on pancreatic histopathology in normal and diabetic rats.** See Figure 2 for groups and treatment. Scale bar = 50 μm.

Histopathological observations were shown in Figure 3. Pancreatic histology of normal control rats was normal throughout the whole study. The appearance of GSPs (250 mg/kg BW)-treated pancreas was similar to that of normal control ones. On the contrary, pancreas showed evidence of severe damage characterized by reduced pancreatic islet area in diabetic control rats. Although 125 mg/kg BW GSPs had no notable effect on the level of damage, the atrophied pancreatic islets were ameliorated in 250 and 500 mg/kg BW GSPs-treated diabetic rats.

### Electron microscopic analysis of pancreatic beta cells

**Figure 4:**
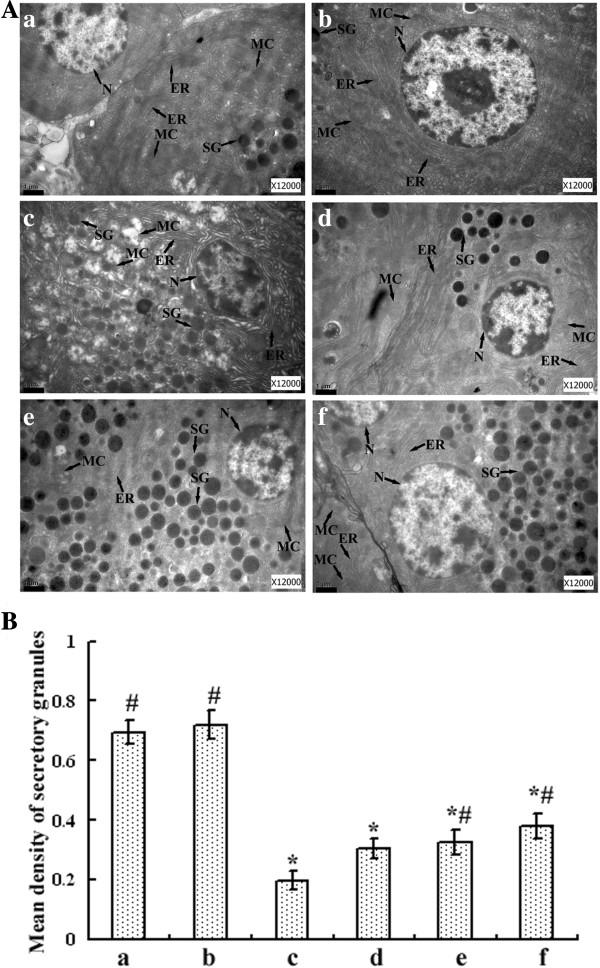
**Electron microscopic studies on pancreatic beta cells of normal and diabetic rats.** Ultrastructural organization of pancreatic beta cells (**A**). Scale bar = 1 μm. ER: endoplasmic reticulum; MC: mitochondria; N: nucleus; SG: secretory granules. Mean density of SG was assessed by Image-Pro Plus 6.0 software and calculated as means ± SEM of 20 determinations in each group (**B**). See Figure 2 for groups and treatment. * P < 0.05 versus vehicle-treated normal rats, and # P < 0.05 versus vehicle-treated diabetic rats.

Pancreatic beta cells store insulin in secretory granules that undergo exocytosis upon glucose stimulation. As depicted in Figure 4, beta cells of normal rats contained secretory granules in the cytoplasm, with moderate homogenous, or slightly heterogenous electron density; meanwhile, the nucleus, mitochondria and ER were normal in beta cells of these rats. However, secretory granules of beta cells of diabetic rats were significantly diluted when compared with that of normal rats (P < 0.001), suggesting immature granules were increased. In addition, pancreatic beta cells of diabetic rats showed pathological alterations, including nuclear condensation, mitochondrial vacuolization, and swelling and dilatation of ER. 250 and 500 mg/kg BW GSPs treatments had obvious beneficial effects on the diluted secretory granules (P = 0.03 and P = 0.003, respectively). The protective effect of GSPs (especially at the dose of 500 mg/kg BW) on diabetic rats was also evident with moderate increases in normal mitochondria, moderate dilatation of ER and the apparently normal architecture of nucleus.

### Effect of GSPs on islet cell apoptosis

**Figure 5:**
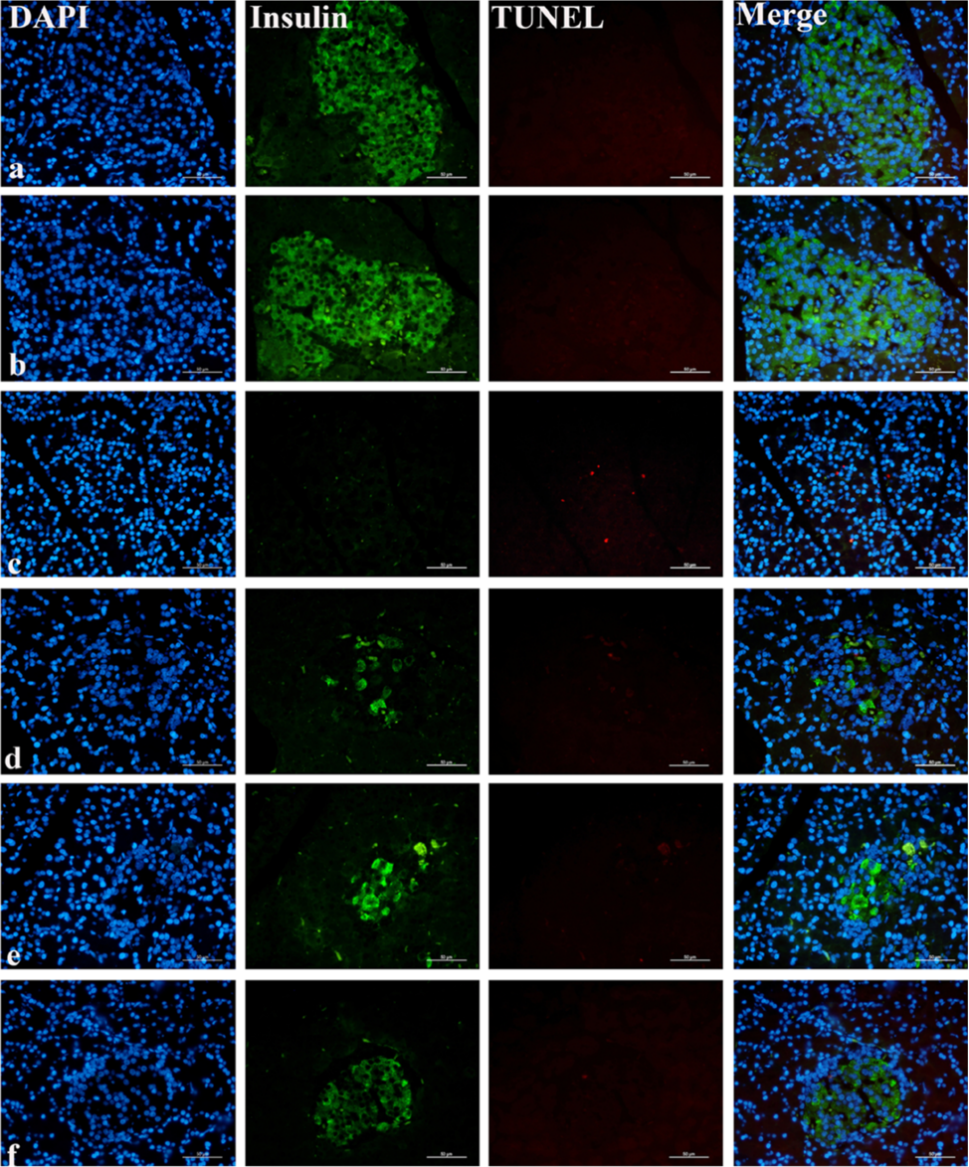
**Effect of GSPs on islet cell apoptosis in normal and diabetic rats.** The slides were visualized by fluorescence microscope. The insulin antibody and fluorescein isothiocyanate (FITC)-labeled secondary antibody were used to probe insulin (green). 4’, 6-diamidino-2-phenylindole (DAPI) was used to visualize nuclei (blue). Apoptotic cells exhibited strong nuclear red fluorescence using regional terminal deoxynucleotidyl transferase-mediated dUTP biotin nick end labeling (TUNEL) staining. See Figure 2 for groups and treatment. Scale bar = 50 μm.

Islet cells undergoing apoptosis were determined by immunostaining for TUNEL assay (Figure 5 and Table 1). There were infrequent apoptotic cells seen in normal and GSPs control islets. However, STZ- and high-carbohydrate/high-fat diet treatment significantly increased the number of TUNEL-positive staining cells compared with normal control ones (P < 0.001). Interestingly, administration of GSPs to diabetic rats reduced TUNEL staining within islet cells in a dose-dependent manner, and 500 mg/kg BW GSPs significantly decreased cell apoptosis when compared with diabetic control rats (P < 0.001).

### Effect of GSPs on insulin expression in pancreatic islets

**Figure 6:**
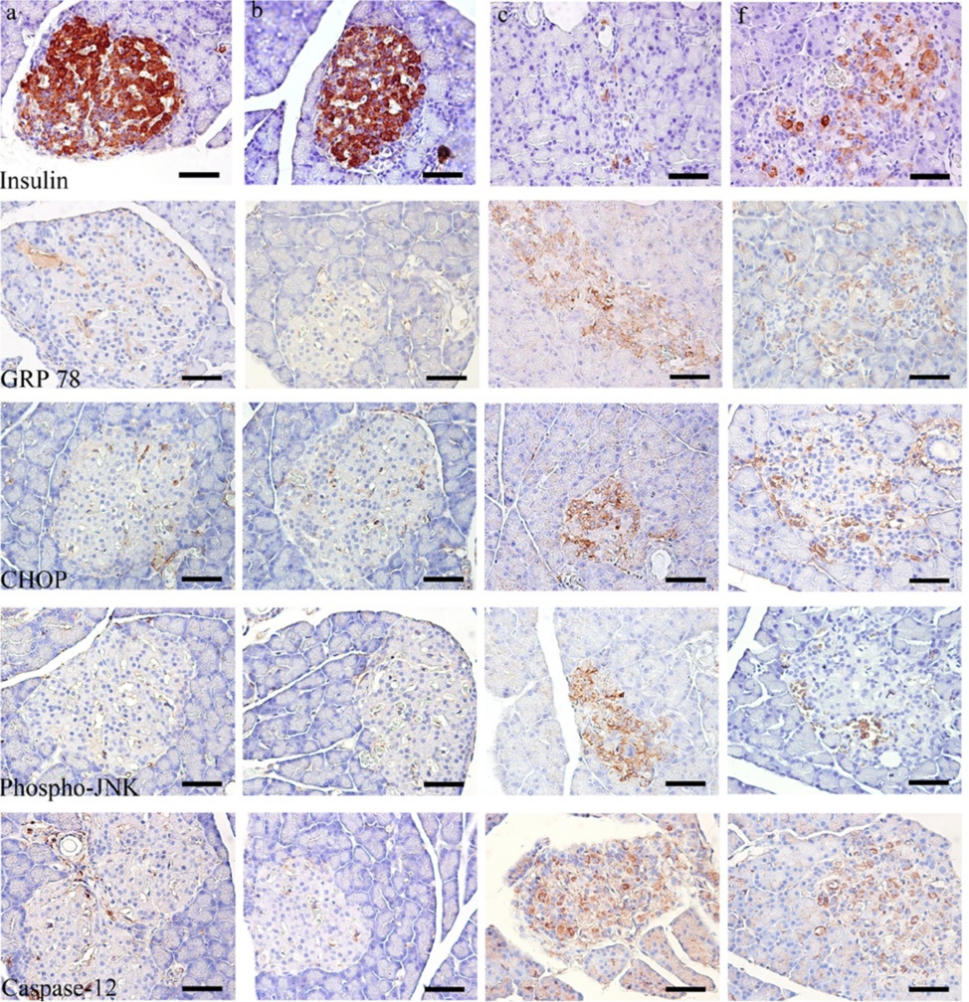
**Immunohistochemistry study on insulin and endoplasmic reticulum stress of islets in normal and diabetic rats.** See Figure 2 for groups and treatment. Scale bar = 50 μm. GRP78: 78-kDa glucose-regulated protein; CHOP: C/EBP homologous protein; Phospho-JNK: phosphorylated Jun N terminal kinase.

Figure 6 and Table 2 demonstrated the level of insulin in pancreatic islets of each group. Healthy pancreatic islets in normal groups exhibited diffused staining with brown or yellow granules. In marked contrast, the insulin expression in diabetic pancreas decreased significantly (P = 0.001) characterized by the depletion of brown or yellow granules. Administration of 500 mg/kg BW GSPs to diabetic rats significantly increased the level of insulin when compared with diabetic control rats (P = 0.018). But 125 and 250 mg/kg BW GSPs treatments had no beneficial effects on the decreased insulin levels (data not shown).

**Table 2:** Effects of GSPs on the expression of insulin and endoplasmic reticulum stress markers {78-kDa glucose-regulated protein (GRP78), C/EBP homologous protein (CHOP), phosphorylated Jun N terminal kinase (Phosphor-JNK) and Caspase-12} of pancreatic islets in normal and diabetic rats

**Parameter (integrated optical density)**	**Normal rats**		**Diabetic rats**	
	**Vehicle**	**GSPs (250 mg/kg BW)**	**Vehicle**	**GSPs (500 mg/kg BW)**
Insulin expression	39674.25 ± 3027.60 ^#^	38034.72 ± 2493.37 ^#^	2337.21 ± 531.25 ^*^	6942.99 ± 896.58 ^* #^
GRP78 expression	1690.20 ± 491.01 ^#^	1762.32 ± 468.30 ^#^	15823.37 ± 2946.95 ^*^	4180.61 ± 384.72 ^*^
CHOP expression	2835.46 ± 711.55 ^#^	2595.55 ± 539.14 ^#^	5725.45 ± 1077.68 ^*^	4642.29 ± 1048.88
Phospho-JNK expression	364.21 ± 109.88 ^#^	395.41 ± 151.75 ^#^	3280.48 ± 661.21 ^*^	682.39 ± 250.81 ^#^
Caspase-12 expression	480.29 ± 134.28 ^#^	518.60 ± 169.85 ^#^	5573.96 ± 1106.86 ^*^	3768.57 ± 773.68

Data were means ± SEM of 50 pancreatic islets of each group.

* designated statistically significant difference from vehicle-treated normal rats, P < 0.05, # designated statistically significant difference from vehicle-treated diabetic rats, P < 0.05.

### Effect of GSPs on ER stress in pancreatic islets

We finally examined the progression of ER stress in pancreatic islets. As can be seen in Figure 6 and Table 2, GRP78, CHOP, phosphorylated JNK and Caspase-12 were all expressed at low levels in both vehicle-treated and GSPs-treated normal rats. However, these ER stress markers were all significantly elevated in pancreatic islets of diabetic animals compared with that in normal control ones (P < 0.05 for each). Interestingly, 500 mg/kg BW GSPs significantly decreased the activity of JNK (P < 0.001), and partly inhibited the protein expressions of GRP78, CHOP and Caspase-12. Nonetheless, 125 and 250 mg/kg GSPs treatments did not significantly inhibit the elevated ER stress markers (data not shown).

## Discussion

It was suggested that extreme nutritional condition was a good way to initiate insulin resistance (IR) [20,21]. At the same time, multi-administration of low dose STZ induced a gradual, autoimmune destruction of beta cells, which might happen in decompensated phase of T2DM [22-24]. In the preliminary experiment, we developed rat models by feeding them with a high-carbohydrate/high-fat diet for 8 weeks accompanying by low dose STZ (20, 25, 30 mg/kg BW) twice injection. Based on our criteria for diabetes (fasting glucose ≥ 250 mg/dl), the successful rate of 25 mg/kg BW STZ group was significantly high and the rats presented a typical characteristic of T2DM as hyperglycemia, IR, and blood lipid disorder, but without decreased serum insulin level (data not shown). So this model was used in subsequent experiments. At the end of the study, diabetic rats still showed symptoms of hyperglycemia, hyperinsulinemia, IR and blood lipid disorder (more details were referred to in our recent report [18]). Concomitantly, pancreatic damage and dysfunction were also existed. Altogether, these results indicated that this stable animal model in the present study might be suitable to investigate the pathogenesis of pancreatic dysfunction and T2DM.

So far, the effects of GSPs have been mainly based on chemically induced type 1 diabetic animals. Among these studies, the results that GSPs had no hypoglycemic effects also existed [25,26]. These contradictory conclusions most likely resulted from multiple factors, including animal species, small sample size, plasma glucose detection methods, changes in dosage and injection techniques of chemical agents, and variation in GSPs dosage and duration. By contrast and in accordance with our data, the administration of proanthocyanidins from other plants (e.g., persimmon peel, cacao liquor and cinnamon bark) were reported to have hypoglycemic activities in type 2 diabetic animal models [27-29]. Interestingly, the present study showed that GSPs exerted ameliorative effects on hyperglycemia in type 2 diabetic animal models. The effects might be accomplished in part by restoration of normal architecture and function of beta cells, as an *in vivo* study based on type 1 diabetic models showed [16].

The remained beta cells mass in diabetic rats places a high demand on the ER for the synthesis of proinsulin. Since proinsulin represents up to 20% of the total mRNA and 30-50% of the total protein synthesis in beta cells [30], misfolded mutant insulin proteins might be a potent cause of ER stress. It was reported that the Akita mouse had a folding mutation in proinsulin that activated the ER stress response, resulting in diabetes with loss of beta cell mass [11]. Our results were in agreement with this report. Although the number of secretory granules in beta cells of diabetic rats did not change significantly, the optical density value of secretory granules were significantly diluted and the insulin expression was significantly decreased when compared with that of normal rats, suggesting that immature insulin or misfolded mutant proinsulin was increased. In addition, it is thought that chronically elevated levels of circulating free fatty acids and glucose are putative mediators of progressive beta-cell dysfunction and death in T2DM [31]. Previous studies showed that ER stress occurred in islets isolated from db/db mice and in pancreas sections of humans with T2DM [10]. Moreover, a requirement for ER stress was previously described for palmitate-mediated beta cell apoptosis *in vitro*[9,10]. In the present study, apart from hyperglycemia and blood lipid disorder (more details were referred to in our recent report [18]), we provided evidence of swollen ER and induction of GRP78, CHOP, JNK and Caspase-12 in diabetic beta cells, which are predominately regulated under conditions of severe and prolonged ER stress. Altogether, these results suggest that misfolded mutant insulin, glucotoxicity and lipotoxicity might induce ER stress, which appears sufficient to cause pancreatic beta-cell dysfunction and death in type 2 diabetic rats.

A key aspect of our work was the indication that GSPs (especially at the dose of 500 mg/kg BW) alleviated ER stress by restoring moderate dilatation of ER and decreasing the expression of GRP78 and the activities of JNK in diabetic pancreas, which might be one of the mechanisms of its protective action. Indeed, ER stress, protein misfolding and oxidative stress are intimately interrelated. On the one hand, cellular reactive oxygen species (ROS) can increase misfolded protein load and deplete glutathione in ER. ROS can also cause ER stress through modification of proteins and lipids that are necessary to maintain ER homeostasis [32]. On the other hand, it is estimated that approximately 25% of the ROS generated in a cell might result from the formation of disulphide bonds in ER during oxidative protein folding [33]. ER stress also increases leak of Ca^2+^ from the ER lumen. Increases in cytosolic Ca^2+^can stimulate mitochondrial ROS production through multiple mechanisms [32]. Subsequently, ROS production can further amplify ER stress and cause cell death. It is notable that pancreatic beta cells are considered especially susceptible to attacks by oxidative stress because of the very low expression of antioxidant enzymes as compared to other tissues. GSPs were reported to possess a broad spectrum of pharmacological, medicinal and therapeutic properties against oxygen free radicals and oxidative stress [34-36]. So it is still possible that GSPs affect ER stress by preventing oxidative stress on diabetic pancreas. It remains to be determined which mechanisms are accountable for the present observations.

## Conclusion

Our results suggested that pancreatic beta-cell dysfunction and death in T2DM might be due, at least in part, to ER stress. GSPs brought about its anti-diabetic effect through normal insulin secretion from the remnant beta cells. GSPs also alleviated ER stress possibly via restoration of moderate dilatation of ER and inhibition of some ER stress markers in diabetic pancreas. This study confirmed and extended data regarding the anti-diabetic potential of GSPs *in vivo* when administered orally to experimentally diabetic rats induced by low dose STZ and a high-carbohydrate/high-fat diet. However, dissection of the contribution of ER stress to pathological conditions is quite challenging because of the lack of specific inhibitors suitable for *in vivo* administration. Further *in vitro* research into the effect of GSPs on pancreatic beta-cell failure and ER stress will contribute to our understanding of the identification of new molecular targets for protection against diabetes.

## Abbreviations

CHOP: C/EBP homologous protein; ER: Endoplasmic reticulum; GRP78: Glucose regulated protein 78; GSPs: Grape seed proanthocyanidins; HOMA-B: Homoeostasis model assessment of beta-cell function; IR: Insulin resistance; JNK: Jun N terminal kinase; OGTT: Oral glucose tolerance test; ROS: Reactive oxygen species; STZ: Streptozotocin; TUNEL: Terminal deoxynucleotidyl transferase-mediated dUTP biotin nick end labeling; UPR: Unfolded protein response.

## Competing interests

The authors declare that they have no competing interests.

## Authors’ contributions

YD and ZZ participated in study design, the animal experiment, data interpretation and manuscript writing. YL gave the original idea and was in charge of the whole trial. XD, YJ, LB and YL assisted with the animal trial and biochemical assays. All authors read and approved the final manuscript.
